# Multivariate Statistical Analysis of Metabolites in *Anisodus tanguticus* (Maxim.) Pascher to Determine Geographical Origins and Network Pharmacology

**DOI:** 10.3389/fpls.2022.927336

**Published:** 2022-06-29

**Authors:** Chen Chen, Bo Wang, Jingjing Li, Feng Xiong, Guoying Zhou

**Affiliations:** ^1^Chinese Academy of Sciences Key Laboratory of Tibetan Medicine Research, Northwest Institute of Plateau Biology, Xining, China; ^2^University of Chinese Academy of Sciences, Beijing, China; ^3^College of Life Science, Qinghai Normal University, Xining, China

**Keywords:** *A. tanguticus*, metabolites, chemometrics analysis, network pharmacology, geographical origins

## Abstract

*Anisodus tanguticus* (Maxim.) Pascher, has been used for the treatment of septic shock, analgesia, motion sickness, and anesthesia in traditional Tibetan medicine for 2,000 years. However, the chemical metabolites and geographical traceability and their network pharmacology are still unknown. A total of 71 samples of *A. tanguticus* were analyzed by Ultra–Performance Liquid Chromatography Q–Exactive Mass Spectrometer in combination with chemometrics developed for the discrimination of *A. tanguticus* from different geographical origins. Then, network pharmacology analysis was used to integrate the information of the differential metabolite network to explore the mechanism of pharmacological activity. In this study, 29 metabolites were identified, including tropane alkaloids, hydroxycinnamic acid amides and coumarins. Principal component analysis (PCA) explained 49.5% of the total variance, and orthogonal partial least-squares discriminant analysis (OPLS–DA) showed good discrimination (R2Y = 0.921 and Q2 = 0.839) for *A. tanguticus* samples. Nine differential metabolites accountable for such variations were identified through variable importance in the projection (VIP). Through network pharmacology, 19 components and 20 pathways were constructed and predicted for the pharmacological activity of *A. tanguticus*. These results confirmed that this method is accurate and effective for the geographic classification of *A. tanguticus*, and the integrated strategy of metabolomics and network pharmacology can explain well the “multicomponent—-multitarget” mechanism of *A. tanguticus*.

## Introduction

Herb have been extensively used worldwide for treating and preventing diverse human diseases and for maintaining health because of their good therapeutic activity (Simpson and Ogorzaly, 2001; Zeng et al., 2007; Chen et al., 2009; Jiang et al., 2010). In ancient times, herbs were used as folk medicines, and herbs are increasingly receiving worldwide attention because of their effect on treating diseases. The therapeutic effects of herbs are derived from the synergistic effect of its metabolites. However, metabolites in herbs differ depending on their geographical origins and influenced by environmental factors, and may influence the quality of herbs (Lukas et al., 2014; Pan et al., 2015; Nehal and Ashaimaa, 2020; Xiong et al., 2021; Rita et al., 2022).

*Anisodus tanguticus* (Maxim.) Pascher, named “Tang Chun Na Bao” in traditional Tibetan medicine, is mainly grown in the Qinghai–Tibet Plateau, China (Wang et al., 2005). *A. tanguticus* is purportedly bitter, warm, analgesic and spasmodic, activating blood to remove stasis, stop bleeding and generate muscles (Zhang and Wang, 2002). *A. tanguticus* is of interest mainly because it has tropane alkaloids, including anisodamine, atropine, scopolamine, and anisodine. These compounds have an anticholinergic effect and are used as analgesics, sedatives, asthma treatment, and antispasmodics (Drager, 2007; Boros et al., 2010; Oniszczuk et al., 2013). However, different *A. tanguticus* have distinct chemical compositions, resulting in diverse quality and health effects. The chemical compositions of *A. tanguticus* are affected by environmental factors, differing in climate factors (such as temperature, precipitation, and sunshine hours), geographical factors (such as altitude and slope), which can affect the growth and metabolites of the plant. Therefore, identifying metabolites from different geographical origins is important for optimizing the development and utility of *A. tanguticus*.

For this reason, the development of powerful analytical methods to assess the geographical origin of herbs is in high demand. Methods including HPLC, GC, HPLC–MS, ICP–MS, NIR, combined with chemometric methods, have recently been used to discriminate the geographical origin of herbal medicines (Yang et al., 2018; Miao et al., 2019; He et al., 2020; Long et al., 2021). Among these techniques, non-targeted metabolite analysis using mass spectrometry (MS) with chemometrics is an emerging approach for food and herb authentication studies due to its several advantages in terms of sensitivity, speed, and high-throughput analysis. In particular, Ultra–Performance Liquid Chromatography Q–Exactive Mass Spectrometer (UPLC–Q–Exactive–MS) has been proven to be a good instrument for metabolomic analysis and has considerable advantages in metabolomics analysis, owing to its high resolution, excellent powerful separation and sensitivity (Chen et al., 2020).

Network pharmacology is a new field of pharmacology, that integrates systems biology, multi–pharmacology, and computational biology to explain the complex pharmacology of herbal medicines (Gong et al., 2022; Wang et al., 2022; Xi et al., 2022). Thus, integrating network pharmacological analysis with metabolites may provide a good way to explain the pharmacological mechanism of *A. tanguticus*.

To our knowledge, there are no reports on discrimination of *A. tanguticus* by metabolites combined with multivariate analysis, and there are no reports explaining the pharmacological mechanism of *A. tanguticus*. The objectives of this study were as follows: (1) to identify the metabolites in *A. tanguticus* by UPLC–Q–Exactive–MS and (2) to develop a multivariate statistical analysis to classify *A. tanguticus* based on geographic origin. (3) establishment of network pharmacology to explain the pharmacological mechanism of *A. tanguticus*.

## Materials and Methods

### Materials

**Figure 1:**
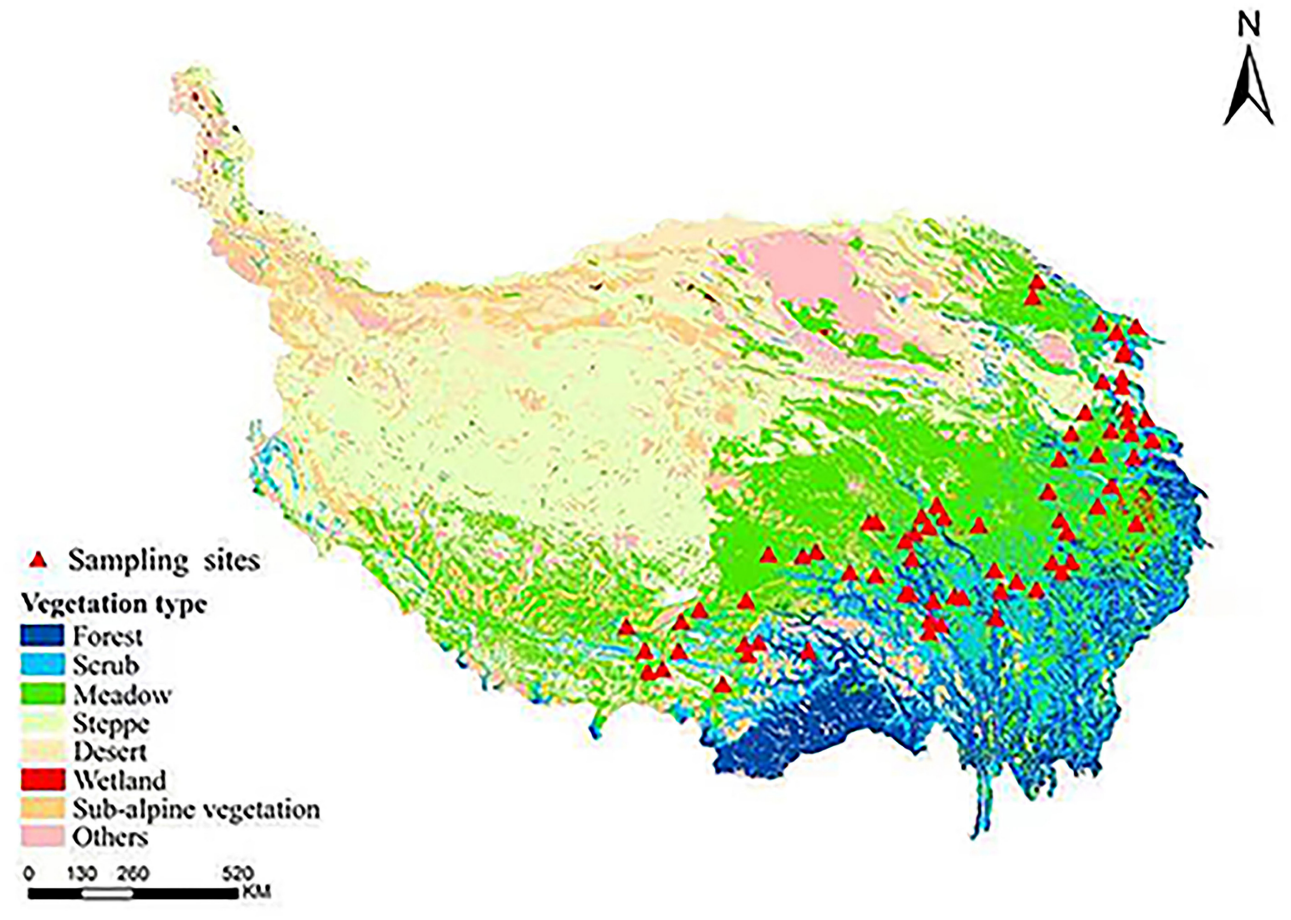
Geographical distribution of the regions of *A. tanguticus*.

Three roots with similar morphological characteristics were randomly selected for sampling. In all, 71 samples of *A. tanguticus* were collected at altitudes ranging from 2,700 m to 4,100 m, longitude ranging from 95° to 102°, and latitudes ranging from 30° to 38° in August 2020 in Qinghai–Tibet Plateau, China (Figure 1). The sampling sites basically covered the entire geographic distribution area of *A. tanguticus* according to the specimen information of *A. tanguticus* was collected from the NSII China National Specimen Resource Platform (http://www.nsii.org.cn/) and China Digital Herbarium (https://www.cvh.ac.cn/). MS grade acetonitrile and formic acid were obtained from Merck.

### Sample Preparation

Fresh roots were carefully cleaned and dried in a hot-air oven (37 ± 2°C). After that, the dried samples were ground as powder followed by sieving with a 100-mesh sifter. 200 mg of sample was transferred to a flask with 10 mL of 80% methanol followed by ultrasonic extraction for 30 min at room temperature. The solution was filtered by a 0.22 μm syringe filter before UPLC–Q–Exactive–MS analysis.

### UPLC–Q–Exactive Mass Spectrometer Conditions

UPLC–Q–Exactive–MS (Thermo Scientific, USA) was used for analysis. The column was a UHPLC column (250 mm × 2.1 mm, 1.8 μm, RP-C_18_). The mobile phases were (a) water with 0.1% (v/v) formic acid and (b) acetonitrile with 0.1% (v/v) formic acid. The optimized elution conditions were as follows: from 5 to 5% A (v/v) at 8 min, from 5 to 10% A (v/v) at 20 min, from 10 to 30% A (v/v) at 30 min, from 30 to 50% A (v/v) at 35 min, and from 50 to 90% A (v/v) at 45 min. The flow rate was 0.2 mL/min. The injection volume was 3 μL. Chromatograms were obtained at 254 nm using photo-diode array. The column temperature was 55°C. The full spectra scanning range of MS was m/z 100–1,500. The optimized parameters were set as follows: electrospray voltage, 3.5kV; vaporizer temperature, 280°C; capillary temperature, 330°C; gas flow, 10 L/min; nebulizer gas pressure, 2.5 bar; collision energy, 50 V.

### Mass Data Processing

Then, the raw data were imported into the Progenesis QI software (Waters, USA). Peak detection, data mining alignment and normalization, and normalized data were subsequently performed. These normalized data were fed into SIMCA 14.1 (Umetrics, Kinnelon, USA) for multivariate analysis. Principal component analysis (PCA) and orthogonal partial least-squares discriminant analysis (OPLS-DA) were performed using SIMCA.

### Network Pharmacology

The molecular targets of the metabolites were limited to Homo sapiens to obtain reliable results. In *A. tanguticus*, molecular targets were identified by SwissTargetPrediction (http://www.swisstargetprediction.ch/). The interactions of targets with high scores (≥0.9) were used to construct network analysis by the STRING database. Gene Ontology (GO) and Kyoto Encyclopedia of Genes and Genomes (KEGG) signaling pathway analyses were performed using the Database of Annotation, Visualization and Integrated Discovery (DAVID, https://david.ncifcrf.gov/).

## Results

### Identification of the Main Metabolites

**Figure 2:**
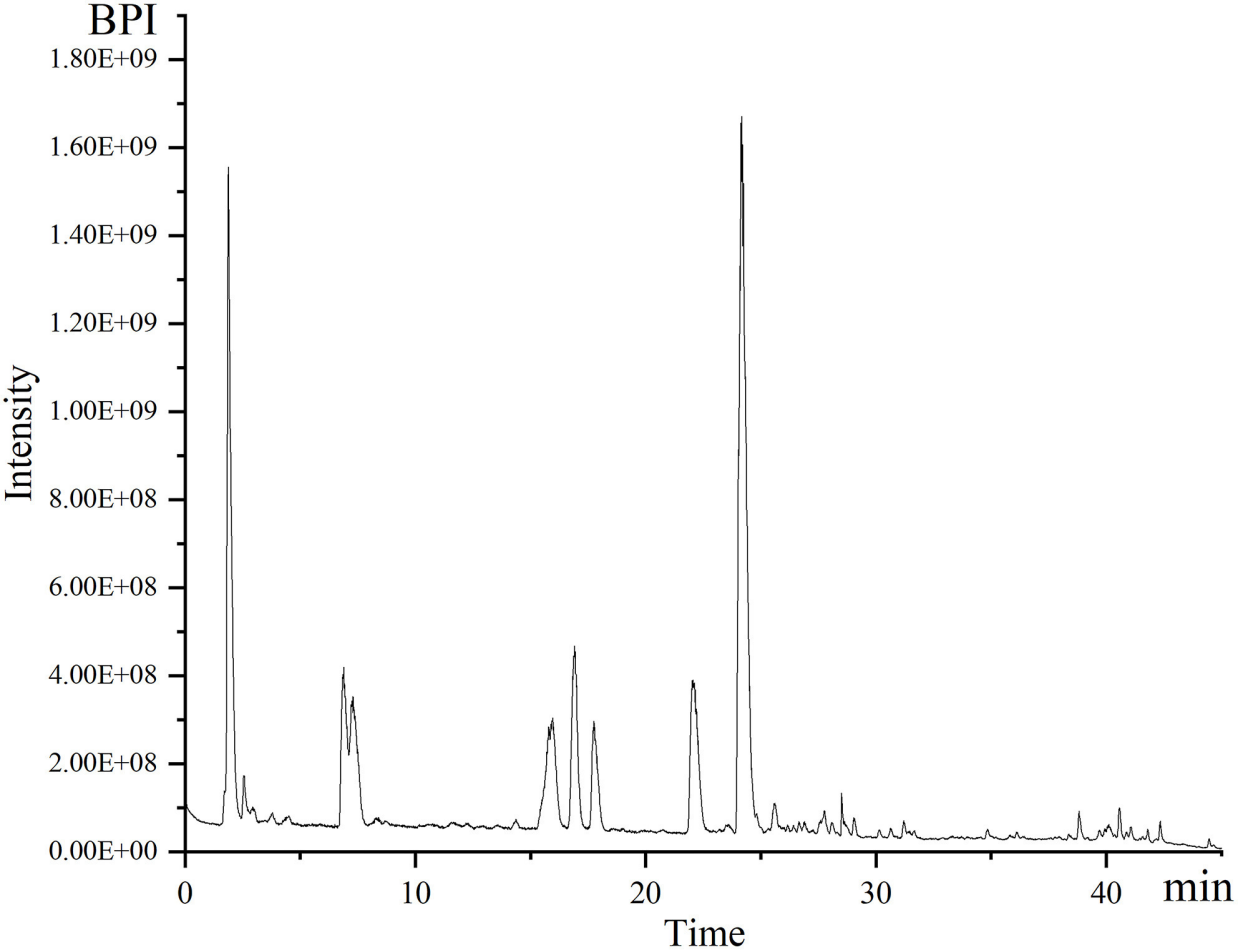
The base peak ions (BPI) chromatogram of *A. tanguticus* by UPLC–Q–Exactive–MS in positive ion mode.

Separation and identification of metabolites from *A. tanguticus* were performed using UPLC–Q–Exactive–MS. Chromatographic profiling provides a relatively complete picture of metabolomic analysis, which is usually used for the identification and authentication of herbs and their products. Figure 2 shows an example of chromatograms of peaks obtained in positive mode. The mass of the parent ion and the fragment ions are obtained for each analyte. Table 1 lists in detail the detected compounds according to their retention time, molecular formula, m/z and formed adducts. A total of 29 compounds were detected and qualified. Five tropane alkaloids (anisodine, convolamine, anisodamine, atropine, noratropine), which have tropane ring characteristics, were identified in our previous study (Chen et al., 2021). Except for five tropane alkaloids, physochlain was identified based its tropane ring characteristics.

**Table 1:** Mass spectrometric data of *A. tanguticus*.

**Peak**	**t_**R**_**	**[M+H]^**+**^ (m/z)**	**Formula**	**Fragment (m/z)**	**Tentative identification**	**Error (mmu)**	
	**(min)**						
1	4.23	268.1039	C_10_H_13_N_5_O_4_	136, 119	Adenosine	−0.48	Standard
2	6.41	322.2119	C_17_H_27_N_3_O_3_	251, 224, 186, 163, 135	*N*-(4-hydroxy-3-methoxy-cinnamoyl) spermidine	−1.92	
3	7.22	320.1494	C_17_H_21_NO_5_	156, 138, 119, 98	Anisodine	0.47	Standard
4	10.51	306.1692	C_17_H_23_NO_4_	281, 224, 193, 169, 147	Convolamine	1.82	
5	11.87	322.1648	C_17_H_27_N_3_O_3_	193, 156, 113	*N*-(4-hydroxy-3-methoxy-cinnamoyl) spermidine	−0.29	
6	14.11	306.1705	C_17_H_23_NO_4_	288, 140, 169	Physochlain	1.68	
7	15.26	304.1541	C_17_H_21_NO_4_	103, 93	Scopolamine	−0.77	Standard
8	16.38	355.1024	C_16_H_18_O_9_	193, 178	scopolin	0.12	Standard
9	16.65	487.1439	C_21_H_26_O_13_	341, 193, 179	fabiatrin	1.28	Standard
10	17.61	306.1704	C_17_H_23_NO_4_	193, 140, 122, 91	anisodamine	1.35	Standard
11	22.32	474.2606	C_25_H_35_N_3_O_6_	222, 236, 165, 123	*N, N*-bis(dihydrocaffeoyl)spermidine	−1.26	(Yang et al., 2020)
12	23.95	276.1597	C_16_H_21_NO_3_	128, 121, 110, 93	Noratropine	1.01	(Chen et al., 2007)
13	24.32	472.2453	C_25_H_33_N_3_O_6_	293, 220, 165, 163	*N*-hydrocaffeoyl-*N*-caffeoyl-spermidine	−0.53	(Yang et al., 2020)
14	24.49	290.1740	C_17_H_23_N_3_O_3_	124, 93	Atropine	0.94	Standard
15	25.40	193.0497	C_10_H_8_O4	133, 122, 94, 77	Scopoletin	0.85	Standard
16	25.78	470.2293	C_25_H_31_N_3_O_6_	380, 290, 220, 141	*N, N*-Dicaffeoyl-spermidine	0.49	(Yang et al., 2020)
17	26.15	488.2748	C_26_H_37_N_3_O_6_	310, 236, 179, 141	*N*-hydroferuloyl-*N*-hydrocaffeoyl-spermidine	1.29	
18	28.12	502.2909	C_27_H_39_N_3_O_6_	249, 236, 177, 137	*N*-hydroferuloyl-*N*-hydroferuloyl-spermidine	0.51	
19	28.67	500.2756	C_27_H_37_N_3_O_6_	234, 177, 145	*N*-feruloyl-*N*-hydroferuloyl-spermidine	0.17	
20	31.89	314.1388	C_18_H_19_NO_4_	177, 121	Feruloyltyramine	0.37	
21	32.46	344.1490	C_19_H_21_NO_5_	177, 145	N-trans-feruloyl-4'-O-methyldopamine	−0.71	
22	37.18	302.3053	C_18_H_39_NO_2_	121	Sphinganine	−0.19	
23	37.75	346.3313	C_20_H_40_O_3_	258, 180, 121	Hydroxyicosanoic acid	−0.78	
24	39.18	330.3370	C_20_H_40_O_2_	121	Phytanic acid	1.04	
25	40.97	274.2443	C_16_H_32_O_2_	256, 106	Butyl laurate	2.99	
26	41.28	318.3004	C_18_H_36_O_3_	290, 274, 256, 242	Hydroxyoctadecanoic acid	0.41	
27	41.69	219.1744	C_15_H_22_O	201, 184	Turmerone	0.27	
28	42.09	412.2096	C17H_34_NO_8_P	184	1,2-Dihexanoyl-sn-glycero-3-phosphoethanolamine	1.04	
29	43.52	496.3395	C24H_50_NO_7_P	301	1-nonadecanoyl-glycero-3-phosphoethanolamine	0.09	

Hydroxycinnamic acid amides are *N*–acylated biogenic amines conjugated with hydroxycinnamic acids via amide bonds, which are most found in *Solanaceae* (Wu et al., 2016). Hydroxycinnamic acid amides are derived from aliphatic polyamines or aryl monoamines conjugated with phenolic acids, especially hydroxycinnamic acids. All amides break along the amide bond producing acylium ions and protonated amines in positive ion mode, which offers more reliable identification due to predictable fragmentation. For example, for peak 13, [M+H]^+^ is at *m/z* 472, and the formula is C_25_H_33_N_3_O_6_. The fragment ion 165 represents the dihydrocaffeyl ion, 163 represents the caffeoyl ion, *m/z* 310 represents [M+H–caffeoy]^+^, and 293 represents [M+H–caffeoy–NH_3_]^+^. This hydroxycinnamic acid amide fragmentation pathway is suited for other hydroxycinnamic acid amides. Therefore, eight hydroxycinnamic acid amide compounds, including *N*–(4–hydroxy−3–methoxy-cinnamoyl) spermidine 1, *N*–(4–hydroxy−3–methoxy–cinnamoyl) spermidine 5, *N, N*–bis(dihydrocaffeoyl) spermidine 11, *N*–hydrocaffeoyl–*N*–caffeoyl–spermidine 13, *N, N*–dicaffeoyl–spermidine 16, *N*–hydroferuloyl–*N*–hydrocaffeoyl-spermidine 17, *N*–hydroferuloyl–*N*–hydroferuloyl–spermidine 18 and *N*–feruloyl–*N*–hydroferuloyl–spermidine 19 were identified according to the MS data in *A. tanguticus* (Vinzenz et al., 2010; Macoy et al., 2015; Yulian et al., 2016; Li et al., 2018; Wang et al., 2018). Hydroxycinnamic acid amides play essential roles in plant growth and developmental processes, such as cell division, root growth and leaf senescence. In addition, it has been reported that polyamines have antitubercular, anti-inflammatory, and α-glucosidase inhibitory activities (Shakya and Navarre, 2006; Li et al., 2014; Sun et al., 2015).

Three coumarin compounds were identified, including scopoletin, scopolin and fabiatrin. Peak 15 was identified to be scopoletin, which had a protonated molecular ion at *m/z* 193.0497 and fragment ions at m/z 178 ([M+H–CH_3_]^+^), 133 ([M+H–NH_3_-CO_2_]^+^) and 122 ([M+H–CH_3_-2CO]^+^). These data matched the published data (Chang et al., 2013). Scopoletin has been reported to possess hypotensive, rheumatic arthritis therapeutic, xanthine oxidase inhibitory, and antioxidant activities (Ojewole and Adesina, 1983; Yun et al., 2001; Shaw et al., 2003). Peak 8 was identified as scopoline, which displayed loses of 162 ([M+H–glucoside]^+^) and 193 (scopoletin). Scopoline has anti-inflammatory, and analgesic effects (Xie et al., 2008; Guo et al., 2010). Peak **9**, identified as fabiatrin, showed the [M+H]^+^ ion at *m/z* 487.1439 (C_21_H_26_O_13_), and its fragment ions at *m/z* 341, 179, and 147 corresponded to the sequential losses of the rhamnose residue and glucose residue, respectively.

### Principal Component Analysis

Unsupervised PCA was performed to assess sample clustering of different geographical origins and processing without providing any prior information in the first phase of the chemometric analysis. PCA can visualize the data in two or three dimensions and can find the relationship between the variables and samples. In the PCA model, the variables contribute similar information and provide unique information concerning the samples. Therefore, PCA can provide useful descriptors in data, so that the results can be used to distinguish samples and explain the cause of variability.

**Figure 3:**
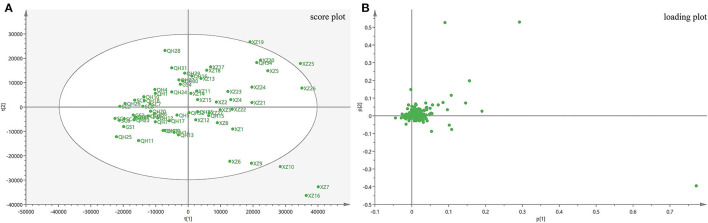
PCA results of *A. tanguticus*. Score plot with the first two principal components of *A. tanguticus* **(A)**, Loading plot of PC1 and PC2 of *A. tanguticus* **(B)**.

The PCA model in this study was obtained from the metabolites in all samples, in which principal component 1 explained 31.4% and principal component 2 explained 18.1% of the total variation. From the scores plot (Figure 3A), it is evident that the samples tended to cluster according to their geographical origins. All samples were classified into two parts along the X axis. The loading scatter plot (Figure 3B) shows how the compounds contribute to the model and which compounds are far away from the center and have a strong impact on the model. The plot shows that atropine, *N*–(4–hydroxy−3-methoxy–cinnamoyl) spermidine, *N, N*–bis(dihydrocaffeoyl) spermidine, *N*–hydrocaffeoyl–*N*–caffeoyl–spermidine, anisodamine and scopoletin have a strong impact on discriminating *A. tanguticus* from various regions. In this study, PCA could distinguish *A. tanguticus* samples from different locations; however, the PCA model did not classify the samples from different geographical origins well. Therefore, OPLS–DA was used to obtain better identification results.

### Orthogonal Partial Least Squares Discriminant Analysis

**Figure 4:**
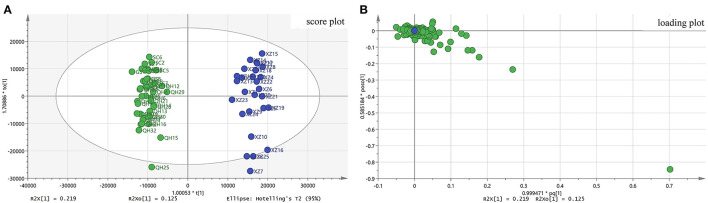
OPLS-DA results of *A. tanguticus*. Score plot from OPLS-DA **(A)**, Loading plot from OPLS-DA **(B)**.

OPLS–DA is a supervised model that was used to differentiate the metabolic profile of the general differences between groups and to find metabolite differences between groups. In the OPLS-DA model, R2Y and Q2 were checked to validate of the accuracy and reliability of the model. When R2Y and Q2 values approach 1, the model should have a better forecast. In this study, the resulting values of R2Y and Q2 of 0.921 and 0.839, respectively, confirmed that the model had goodness of fit and predictability. The classification can be visualized in the scores chart in the OPLS–DA model shown in Figure 4. From Figure 4A, two parts were classified by the Y axis: one part of the samples collected from XZ and the other part of the samples collected from non–XZ. The loading scatter plot (Figure 3B) shows that atropine, *N*–(hydroxy−3-methoxy–cinnamoyl) spermidine, physochlain, anisodamine and scopoletin have a strong impact on discriminating *A. tanguticus* from various regions. The OPLS–DA results showed that the metabolites from different parts were different and influenced by environmental factors. Previous studies have indicated that compounds are associated with season, altitude, or some climate factors (Sun et al., 2016; Lu et al., 2017; Wu et al., 2019). In addition, permutation tests (*n* = 200) were performed to evaluate whether the discriminant models were overfitting the data (Belmonte-Sánchez et al., 2019).

Potential characteristic compounds for separation of the geographical origins of *A. tanguticus* were obtained by filtering with VIP>1, and *p* < 0.05 in the statistical analysis following the *S*-plot. From the results of the *S*-plot, nine compounds *N*-(4-hydroxy-3-methoxy-cinnamoyl) spermidine 1, anisodine 3, fabiatrin 9, *N, N*-bis (dihydrocaffeoyl) spermidine 11, scopoletin 15, *N, N*-dicaffeoyl-spermidine 16, *N*-hydroferuloyl-*N*-hydrocaffeoyl-spermidine 17, *N*-hydroferuloyl-*N*-hydroferuloyl -spermidine 18 and *N*-feruloyl-*N*-hydroferuloyl-spermidine 19 are considered great importance for geographical origin separation.

### Correlation Analysis

**Figure 5:**
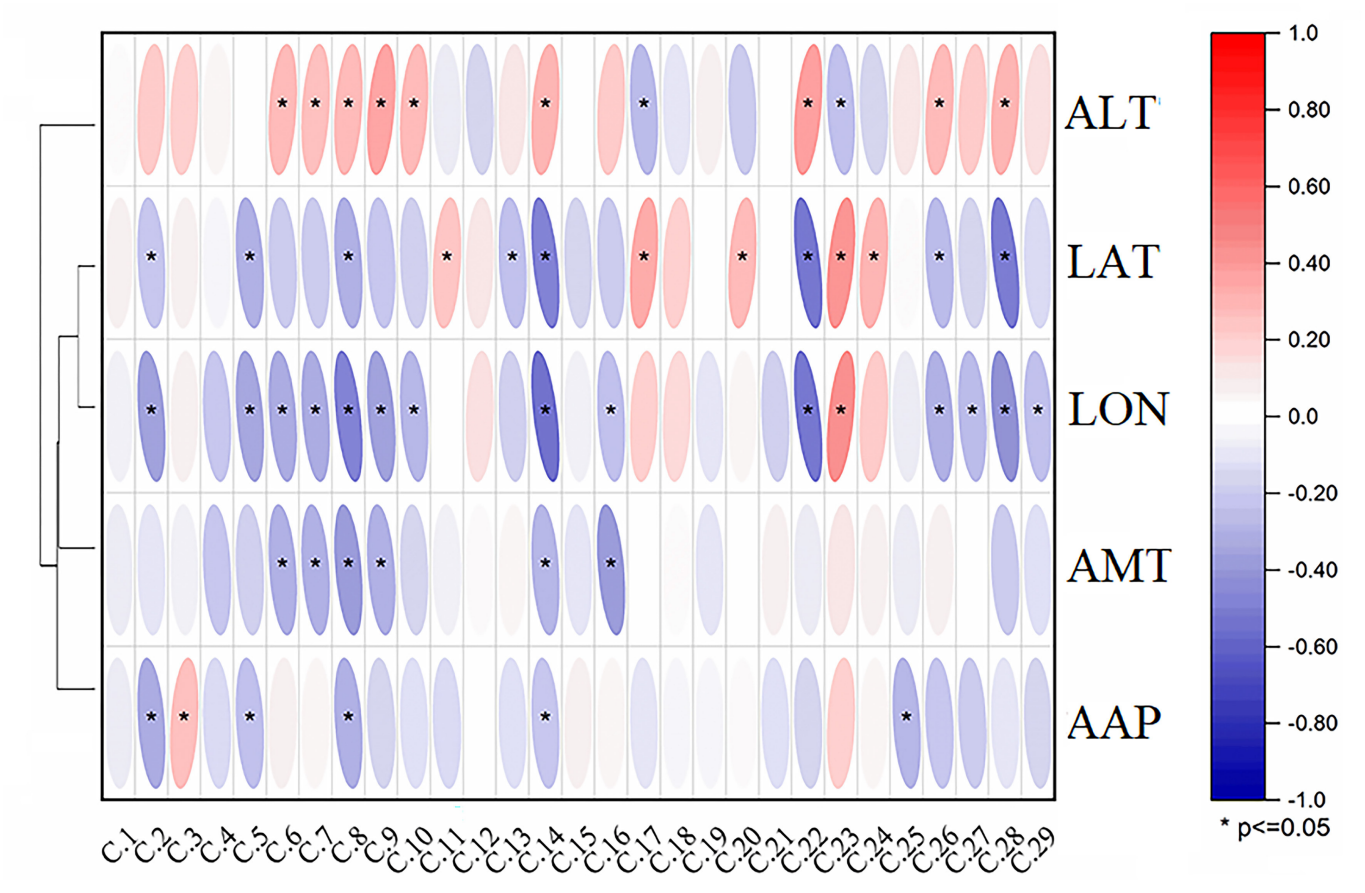
Correlation analysis results of metabolites in *A. tanguticus* and environmental factors [altitude (ALT), longitude (LON), latitude (LAT), annual mean temperature (AMT) and annual average precipitation (AAP)].

To study the effects of different environmental factors on the secondary metabolites of *A. tanguticus*, Pearson's correlation analysis between environmental factors and the relative intensity of 29 compounds in *A. tanguticus* samples is shown in Figure 5, and the relationships between the relative concentrations of the compounds and altitude (ALT), longitude (LON), latitude (LAT), annual mean temperature (AMT) and annual average precipitation (AAP) were determined. The obtained results showed that ALT had significantly negative correlations with compounds 17 and 23 (*p* < 0.05), and significantly positive correlations with compounds 6, 7, 8, 9, 10, 14, 22, 26 and 28 (*p* < 0.05). LON had significantly negative correlations with compounds 2, 5, 6, 7, 8, 9, 10, 14, 16, 22, 26,27, 28 and 29 (*p* < 0.05) and significantly positive correlations with compound 23 (*p* < 0.05). LAT had significantly negative correlations with compounds 2, 5, 8, 9, 13, 14, 22, 26 and 28 (*p* < 0.05) and significantly positive correlations with compound 11, 17, 20, 23 and 24 (*p* < 0.05). AMT was significantly negatively correlated with compounds 6, 7, 8, 9, 14 and 16 (*p* < 0.05); AAP was negatively correlated with 2, 5, 8, 14 and 25 (*p* < 0.05), and it was positively correlated with compound 3 (*p* < 0.05). The environmental factors LAT, LON, AMT and AAP were included in a cluster, and ALT was clustered. Figure 5 shows that LAT, LON, AMT and AAP exhibited significantly negative correlations with most compounds, and ALT showed significant positive correlations with most compounds.

### Network Pharmacology

A total of 19 components were selected as candidate compounds because the compounds are polar compounds that can be extracted by traditional Chinese decoction methods. A total of 829 molecular targets were obtained from SwissTargetPrediction (He et al., 2021). Next, the identified targets were converted into an official gene symbol (Du et al., 2020). Finally, 13 gene clusters and 12 core genes were established after integrating the results.

**Figure 6:**
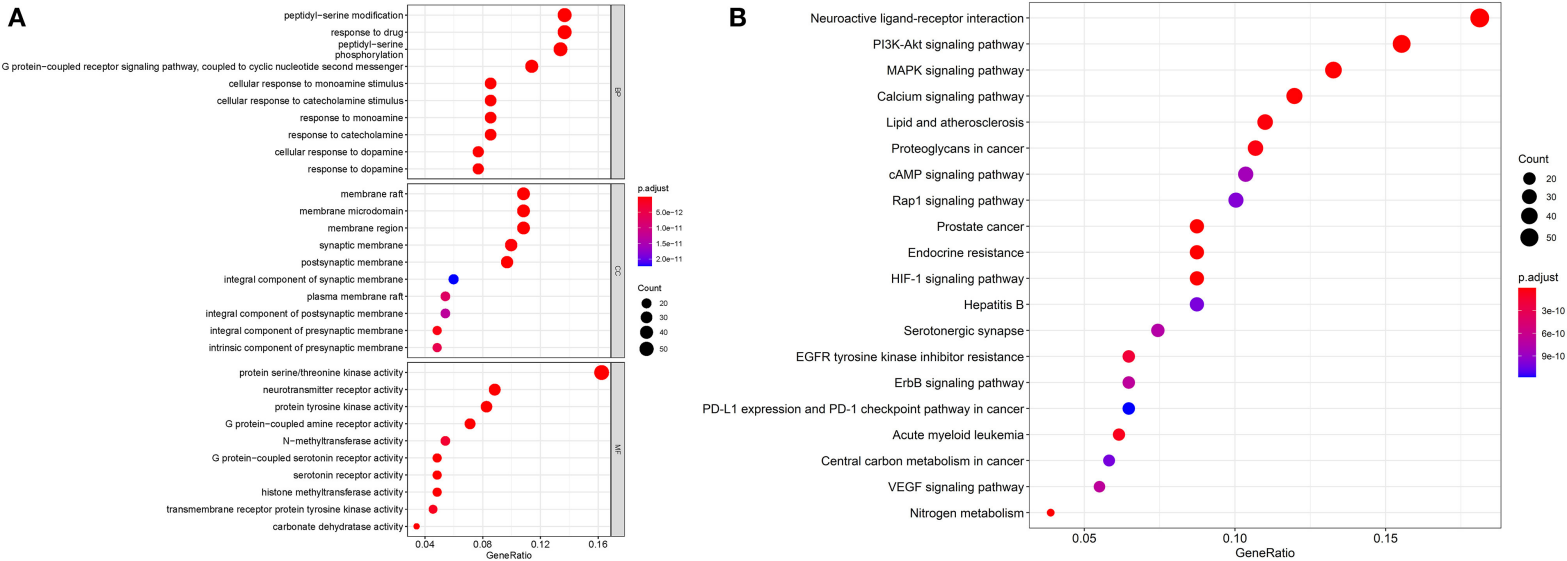
The results of bioinformatics analysis. GO enrichment analysis of targets **(A)**, KEGG pathway enrichment analysis **(B)**.

DAVID was used for GO enrichment. The top 10 terms in the cellular component (CC), biological process (BP), and molecular function (MF) categories were selected and are shown in Figure 6A. BP-related items mainly involve peptidyl–serine modification and peptidyl–serine phosphorylation, response to drug and so on. CC-related items mainly involve membrane rafts, membrane microdomains, membrane regions and so on. MF mainly involve protein serine/threonine kinase activity, neurotransmitter receptor activity, protein tyrosine kinase activity and so on. A total of 163 KEGG pathways (*p* ≤ 0.05) were obtained. The top 20 pathways included the MAPK signaling pathway, PI3K-Akt signaling pathway, and neuroactive ligand–receptor interaction (Figure 6B).

**Figure 7:**
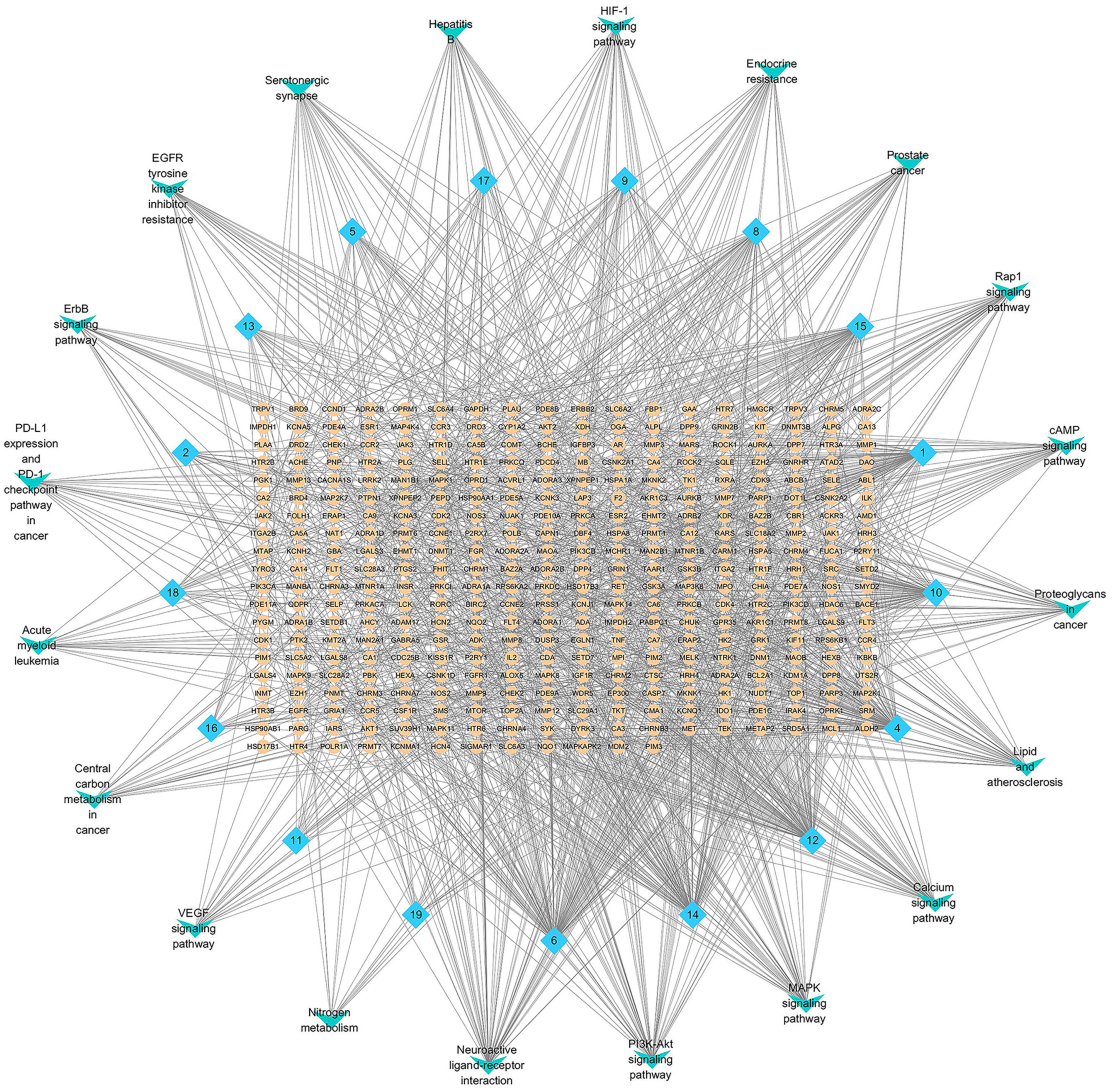
Network pharmacology analysis of difference metabolites in *A. tanguticus*. (Blue is the compound, yellow is the target of the compound, green is the top 20 prominent pathways).

As shown in Figure 7, the network of the compounds, targets and signal path was constructed, where blue is the compound, yellow is the target of the compound, and green is the top 20 prominent pathways. Network pharmacology can provide further mechanisms of the pharmacological activity of *A. tanguticus*.

## Discussion

The eastern Himalayas–Hengduan Mountains region in the southeast of the Qinghai–Tibet Plateau is one of the important origin areas of alpine plants (Wu, 1988; Sun and Zhou, 1996). According to the two classes of samples in the OPLS–DA model and the Figure 1, there may be two routes of geographical migration of *A. tanguticus*. One of the routes is to spread to the western part of the Qinghai-Tibet Plateau through the Brahmaputra River valley, where the water vapor of the Tibetan Plateau is mainly derived from the Indian Ocean; its water vapor is mainly transported to the interior of the plateau through the Valley of the Brahmaputra River, and the strong air currents provide power for the spread of seeds and pollen of plants, thus changing the modern genetic pattern of some alpine plants. The other geographical migration is to the northern part of the Qinghai–Tibet Plateau. When the Quaternary Ice Age arrived, populations on the plateau retreated to refugia in the southeastern plateaus. When the ice age ended, the populations within the shelter were more likely to migrate vertically due to the barrier of the tall mountains, while the pioneer populations at the edge of the shelter were most likely to migrate north to low elevations. Different geographical migrations resulted in different chemical compositions of plants, resulting in XZ samples and other samples being separated.

Multivariate analysis showed that the XZ samples of *A. tanguticus* and other samples could be divided, indicating that there were clear differences in secondary metabolites between the two regions, and these differences were often caused by geographical factors and environmental factors. Through the analysis of geographical factors and climatic factors in the two groups of samples, it could be seen that the average altitude of the samples in XZ is 4,042 m, while the average altitude of the samples in non–Tibet regions is 3,474 m. The average temperature of the samples in Tibet was −0.69°C, while the average temperature of the non-Tibet samples was 0.96°C; the average annual precipitation of the samples in Tibet was 494 mm, and the average annual precipitation of the samples in the non–Tibet region was 555 mm. According to the secondary metabolite resource acquisition hypothesis (Byers, 2000), plant growth will be slow in harsh environments, but secondary metabolites will increase. The Tibetan samples were in a harsher environment, which made the secondary metabolites of the Tibetan samples more helpful to increase the secondary metabolites, thus causing geographical differences between the Tibetan region and the non–Tibetan regions.

As a comprehensive environmental factor, altitude has a regular effect on ecological factors such as temperature, humidity, light, ultraviolet radiation, and CO_2_ concentration, and the long-term adaptation of these ecological factors makes the plants growing at high altitudes form a unique physiological and ecological adaptation mechanism, thus affecting the growth and distribution of plants. As the altitude increases, the temperature decreases, oxygen decreases, and the environment becomes harsher, which may increase the secondary metabolites according to the metabolite resource acquisition hypothesis. This observation confirms our results that most secondary metabolites are positively correlated with altitude. The same results were found for the relationships between temperature and secondary metabolites and between precipitation and secondary metabolites. Higher temperatures and more precipitation make the environment better but are not conducive to the accumulation of secondary metabolites. This observation confirms our results that most secondary metabolites are negatively correlated with temperature and precipitation. However, the correlation analysis shows the relationship of variables, and how environmental factors affect the metabolite is unknown. Thus, further research is needed to show how environmental factors affect the metabolites in *A. tanguticus*.

*A. tanguticus* is a traditional Tibetan medicinal herb that has analgesic effects. The MAPK signaling pathway and PI3K–Akt signaling pathway which are classical inflammatory signaling pathways, were identified in network pharmacology. The PI3K family is a class of protein kinases that are key signaling molecules for life activities. Akt is a PI3K downstream target protein, Akt activation initiates the downstream inflammatory response, and the MAPK signaling pathway can activate and promote the expression of inflammatory factors. It is likely that through these two signaling pathways it exerts anti-inflammatory effects, thus exerting analgesic and analgesic effects.

Cancer signals or signaling pathways that are closely related to cancer, such as prostate cancer, proteoglycans in cancer, EGFR tyrosine kinase inhibitor resistance, VEGF signaling pathway, ErbB signaling pathway, central carbon metabolism in cancer, PD–L1 expression, PD−1 checkpoint pathway in cancer, MAPK signaling pathway and PI3K–Akt signaling pathway were found in network pharmacology, which showed that *A. tanguticus* could have anticancer activity and could be used to develop anticancer activity drug.

Tropane alkaloids in *A. tanguticus* have pharmacologically active and toxicological substances; the symptoms of poisoning of tropane alkaloids act on paralyzing the parasympathetic nerves and produce symptoms of muscarinic poisoning. Neuroactive ligand–receptor interaction, calcium signaling pathway, serotonergic synapse, cAMP signaling pathway and MAPK signaling pathway which realize the toxicological and pharmacological effects were found in network pharmacology. After acetylcholinesterase is inhibited, the increase in acetylcholine excites the sympathetic ganglia, releasing epinephrine and norepinephrine, thus giving *A. tanguticus* a medicinal effect. Increasing the dose leads to vasospasm, destruction of basal ganglia, destruction of the balance of dopamine and acetylcholine, neurotoxicity, triggering a large amount of Ca^2+^ ion inflow, muscle contraction and relaxation, and other biochemical reactions, thereby causing human poisoning. Due to the limitations of this study, the dose relationship between the toxicity and pharmacodynamic activity of tropane alkaloids could not be fully elucidated, and the upregulation and downregulation relationship between the expression of key genes of toxicity and pharmacodynamic activity could not be clarified.

## Conclusions

In this study, untargeted metabolomics using UPLC–Q-Exactive–MS combined with multivariate analysis for the discrimination of *A. tanguticus* samples from different geographical origins was developed. Twenty–nine compounds, including tropane alkaloids, hydroxycinnamic acid amides and coumarins were identified. The metabolites could be used to discriminate samples from different geographical origins. Network pharmacology analysis revealed significant involvement of neuroactive ligand–receptor interaction, the PI3K–Akt signaling pathway, and the MAPK signaling pathway in *A. tanguticus*. which explains the pharmacological activity of *A. tanguticus*. Taken together, the results of this study provide a method to determine geographical origins using combined metabolites and multivariate statistical analysis, to explore pharmacological activity using combined metabolites and network pharmacology.

## Data Availability Statement

The original contributions presented in the study are included in the article/Supplementary Material, further inquiries can be directed to the corresponding author/s.

## Author Contributions

GZ designed the experiments. CC, JL, and BW established and validated the methods. CC and FX were involved in writing of the manuscript. All authors read and approved the final manuscript.

## Funding

This work was supported by Qinghai Science and Technology Achievement Transformation Project (2021-SF-149), Kunlun Talents, High Innovation and Entrepreneurship Talents of Qinghai Province, Joint Grant from Chinese Academy of Sciences-People's Government of Qinghai Province on Sanjiangyuan National Park (LHZX-2020-09), and Key deployment project of Chinese Academy of Sciences (ZDRW-ZS-2020-2).

## Conflict of Interest

The authors declare that the research was conducted in the absence of any commercial or financial relationships that could be construed as a potential conflict of interest.

## Publisher's Note

All claims expressed in this article are solely those of the authors and do not necessarily represent those of their affiliated organizations, or those of the publisher, the editors and the reviewers. Any product that may be evaluated in this article, or claim that may be made by its manufacturer, is not guaranteed or endorsed by the publisher.
