# Supplementary material for: Multivariate Statistical Analysis of Metabolites in Anisodus tanguticus (Maxim.) Pascher to Determine Geographical Origins and Network Pharmacology
Source: Front Plant Sci. 2022 Jun 29;13:927336. doi: 10.3389/fpls.2022.927336 (PMC9277180; doi:10.3389/fpls.2022.927336)
Supplement: Supplementary file 1 [file Image_1.pdf]

Supporting Information

Multivariate statistical analysis of metabolites in *Anisodus tanguticus* (Maxim.) Pascher to determine geographical origins and network pharmacology

Chen Chen [1, 3], Bo Wang [1, 3], Jingjing Li [2], Feng Xiong [1], Guoying Zhou [1*]

[1] *Chinese Academy of Sciences Key Laboratory of Tibetan Medicine Research, Northwest Institute of Plateau Biology, Xining, China*

[2] *Qinghai Normal University, Xining, China.*

[3] *University of Chinese Academy of Sciences, Beijing, China*

* **Correspondence**: Guoying Zhou, PhD, CAS Key Laboratory of Tibetan Medicine Research, Northwest Institute of Plateau Biology, 23# Xinning Road, Xining, China.

Email: zhougy@nwipb.cas.cn

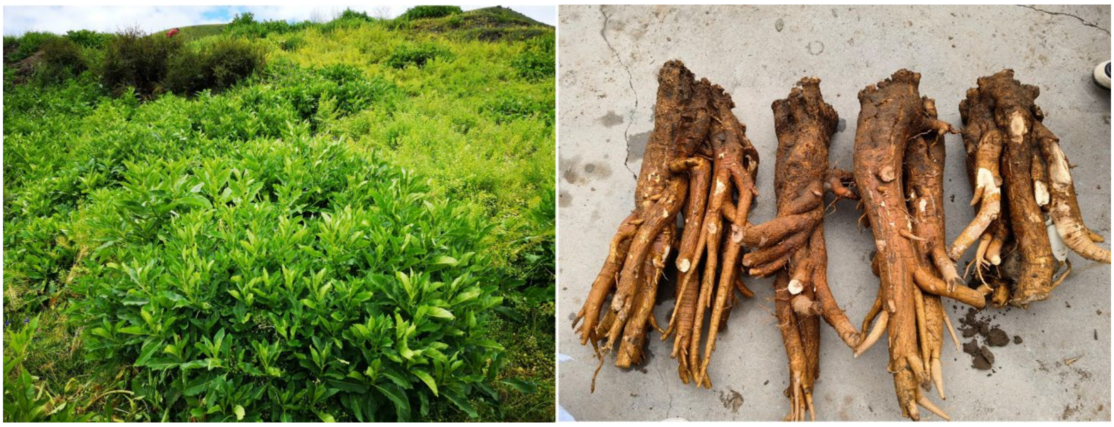

Figure S1 The plant and roots of *A. tanguticus*
